# Modest volcanic SO_2_ emissions from the Indonesian archipelago

**DOI:** 10.1038/s41467-022-31043-7

**Published:** 2022-06-11

**Authors:** Philipson Bani, Clive Oppenheimer, Vitchko Tsanev, Bruno Scaillet, Sofyan Primulyana, Ugan Boyson Saing, Hilma Alfianti, Mita Marlia

**Affiliations:** 1 Laboratoire Magmas et Volcans, Université Blaise Pascal-CNRS-IRD, OPGC, 63170 Aubière, France; 2 Centre IRD de la Nouvelle-Calédonie, 101, Promenade Roger Laroque, BP A5, 98848 Nouméa Cedex, Nouvelle-Calédonie, France; 3 Department of Geography, University of Cambridge, Downing Place, Cambridge, CB2 3EN UK; 4 Institut des sciences de la Terre d’Orléans, Université d’Orléans-CNRS-BRGM, 1a rue de la Férollerie, 45071 Orléans, France; 5 Center for Volcanology and Geological Hazard Mitigation, Jl. Diponegoro No. 57, Bandung, 40122 Indonesia

**Keywords:** Volcanology, Geochemistry

## Abstract

Indonesia hosts the largest number of active volcanoes, several of which are renowned for climate-changing historical eruptions. This pedigree might suggest a substantial fraction of global volcanic sulfur emissions from Indonesia and are intrinsically driven by sulfur-rich magmas. However, a paucity of observations has hampered evaluation of these points—many volcanoes have hitherto not been subject to emissions measurements. Here we report new gas measurements from Indonesian volcanoes. The combined SO_2_ output amounts to 1.15 ± 0.48 Tg/yr. We estimate an additional time-averaged SO_2_ yield of 0.12-0.54 Tg/yr for explosive eruptions, indicating a total SO_2_ inventory of 1.27-1.69 Tg/yr for Indonesian. This is comparatively modest—individual volcanoes such as Etna have sustained higher fluxes. To understand this paradox, we compare the geodynamic, petrologic, magma dynamical and shallow magmatic-hydrothermal processes that influence the sulfur transfer to the atmosphere. Results reinforce the idea that sulfur-rich eruptions reflect long-term accumulation of volatiles in the reservoirs.

## Abstract (web summary)

Indonesia is the country with the most active volcanoes, several of which are renowned for climate-changing eruptions. Here the authors show a rather moderate sulfur emissions budget and reinforce the idea that sulfur-rich eruptions reflect long-term accumulation of volatiles in the reservoirs.

## Introduction

While not the most abundant species in volcanic gases, sulfur dioxide (SO_2_) is the easiest to measure remotely with the aim of deriving a flux. This owes principally to its absorption of ultraviolet (UV) light, enabling daytime spectroscopic measurements from the ground, air and space^1^. Measurements of SO_2_ flux are a cornerstone of volcano monitoring and contribute to the understanding volcanic degassing. They permit the calculation of fluxes of other volcanic gas species (*X*) from measurements of their ratios to sulfur dioxide (*X*/SO_2_) and underpin global inventories of volcanic gas emissions to the atmosphere. In this respect, SO_2_ is particularly important given its roles in atmospheric chemistry and radiation^2^.

Advances in satellite remote sensing of SO_2_ in both ultraviolet and infrared wavebands are adding to our knowledge^3,4^ and the proliferation of compact UV spectrometers and cameras^5–7^, is enabling measurements at less accessible volcanoes. However, the compilation of global inventories still faces numerous challenges, including temporal and spatial data gaps, measurement uncertainties, the presence of multiple sulfur species (including S, H_2_S and H_2_SO_4_) in volcanic emissions, and the challenges of processing large volumes of data.

**Fig. 1:**
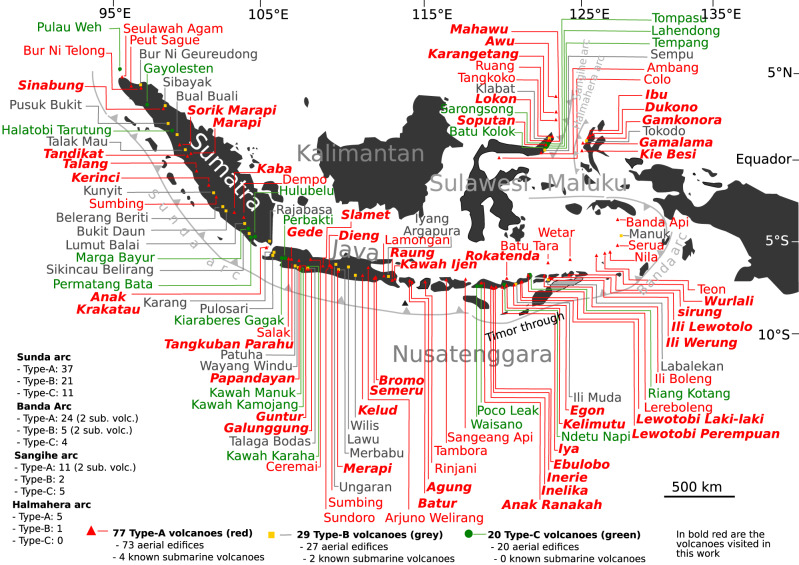
Indonesian active volcanoes. The distribution of the 126 active volcanoes across the archipelago of Indonesia, including 120 aerial and six known submarine edifices (not shown on the map). 77 are classified as Type-A (red triangles), 29 as type-B (yellow squares) and 20 as type-C (green circles) The volcanoes visited in this work are highlighted in red-bold-italic.

One notable lacuna in SO_2_ inventories is the Indonesian archipelago. According to Siebert et al.^8^, there are 78 historically active volcanoes in Indonesia, i.e., those with at least one historically-recorded eruption. But such a definition finds its limit in Indonesia where the documentary record is incomplete and traditional knowledge lost or not fully integrated into scientific records. A more complete inventory of the Indonesian volcanoes can be found in the “Badan Geologi” database^9^ which lists 126 active volcanoes (including six submarine edifices). They are subdivided into 77 type-A volcanoes, which have experienced at least one increase in magmatic and/or phreatic activity since 1600; 29 type-B volcanoes with solfataric and/or fumarolic manifestations but no eruption since 1600; and 20 type-C, which are solfataras and/or fumarole fields lacking a defined volcanic edifice (Fig. 1 and Table 2). These volcanoes fall within four distinct arcs: Sunda, Banda, Sangihe, and Halmahara (Fig. 1).

In the first global compilations of the volcanic SO_2_ budget, the Indonesian contribution was unspecified^10^. Subsequently, over four decades, new observations have furnished estimates of annual SO_2_ inventories for Indonesia (Table 1). These have varied considerably, beginning with the work of Le Guern^11^, who estimated 0.07 Tg /yr (representing just 0.15% of the global volcanic SO_2_ budget) compared with a more recent estimate of Carn et al.^3^ of 2.2 Tg SO_2_ /yr (representing 9.5% of the global volcanic budget). Note that these and intervening works have also reported disparate figures for the global total (Table 1), which is unsurprising given the different datasets (and their timespans) and methods employed. Despite developments in SO_2_ sensing, hitherto only a fraction of the more than 100 Indonesian volcanoes classified as active has been subject to SO_2_ flux measurement campaigns.

**Table 1:** Reported global volcanic SO_2_ inventories and contribution from Indonesian volcanoes.

Authors	Total volcanic SO_2_ (Tg/yr)	Method(s)	Contribution from Indonesian volcanoes
Le Guern, 1982^11^	50.0	Using Correlation Spectrometer (COSPEC) data from ref. ^50^, and extrapolating to a larger number of active volcanoes in different geodynamic provinces.	0.073 Tg yr^−1^ from Indonesia (Merapi volcano was considered as the main degassing source).
Spiro et al., 1992^51^	19.2	Based on plume size, following ref. ^52^ and referring to volcanism in 1964–1972 and 1980: 28% of the SO_2_ emission budget from passive degassing, assuming 61% from eruption to troposphere and 11% to the stratosphere.	0.41 Tg yr^−1^ attributed to Indonesian volcanoes.
Andres and Kasgnoc, 1998^53^	13.4	From a compilation of S fluxes in 214 published references, personal comm., and conference presentations. Electronic mail messages were sent to the VOLCANO list for data discussion with volcanologists and atmospheric scientists. Two categories were distinguished: continuously (49) and sporadically erupting (25) volcanoes.	Indonesian volcanoes contributed to only 0.10 Tg yr^−1^ (four volcanoes were considered, including Merapi, Tangkuban parahu, Bromo and Slamet).
Halmer et al., 2002^54^	15.0–21.0	Considering the SO_2_ emissions of 50 volcanoes recorded by the Total Ozone Mapping Spectrometer (TOMS) and COSPEC, then extrapolated to 310 unmeasured volcanoes based on the VEI-SO_2_ relationship, magma composition, tectonic setting and the state of activity.	2.1–2.6 Tg yr^−1^ attributed to Indonesian subduction zone.
Shinohara, 2013^55^	19.8	Based on a literature review: 76 persistently degassing volcanoes release an estimated 18.5 Tg/yr of SO_2_ and the time-averaged annual SO_2_ flux from explosive eruption (1.3 Tg/yr) is obtained based on VEI-SO_2_ emission correspondence.	0.1 Tg from Indonesia, four volcanoes were considered: Merapi, Tangkubanparahu, Slamet and Bromo.
Carn et al., 2017^3^	23.0	Based on OMI data spanning 2005–2015 and focused on passive degassing from 91 volcanoes worldwide.	2.2 Tg from Indonesia. 20 volcanoes were considered: Dukono, Bromo-Semeru, Lewotolo-Batu Tara, Ijen- Raung, Sirung, Sinabung, Karangetang, Krakatau, Kerinci, Slamet, Lokon, Ebulobo, Rinjani, Sangeang api, Paluweh Marapi and Merapi (from the highest to the lowest SO_2_ emission).

Here we present a new inventory of volcanic SO_2_ emissions for Indonesia based on portable ground-based remote sensing instruments and systematic program of fieldwork observations. We focus our efforts on the subaerial type-A volcanoes with passive degassing, which we consider based on field observation to be the main volcanic degassing sources in Indonesia (Table 2 and Fig. 1). We use the term ‘passive’ to refer to the style of gas emission so as to distinguish it from larger, sporadic explosive emissions, though the term can encompass a wide range of sources from magmatic to fumarolic. We evaluate the factors influencing the variations identified between volcanoes and between sub-regions of Indonesia and consider the total SO_2_ emission rate for the archipelago in the global context.

**Table 2:** The distribution of the Indonesian active volcanoes per type (A, B or C), region and arcs.

	Type-A	Type-B	Type-C		Type-A	Type-B	Type-C
Sunda Arc	**Sumatra**			Banda Arc	**Flores-Lembata-Pantar**		
	Seulawah Agam	Bur Ni Geureudong	Pulau Weh		Anak Ranaka	Ili Muda	Waisano
	Peut Sague	Sibayak	Gayolesten		Inelika	Labalekan	Poco Leak
	Bur Ni Telong	Pusuk Bukit	Halatobi Tarutung		Inerie	Yersey (sub. volc.)	Ndetu Napi
	Sinabung	Bual Buali	Halubelu		Ebulobo		Riang Kotang
	Sorik Marapi	Talak Mau	Marga Bayur		Iya		
	Tadikat	Kunyit	Permatang Bata		Kelimutu (Paluweh)		
	Marapi	Bemerang Beriti			Rokatenda		
	Talang	Bukit Daun			Batu Tara		
	Kirinci	Lumut Balai			Egon		
	Sumbing	Sikicau Belirang			Lewotobi Laki-Laki		
	Kaba	Rajabasa			Lewotobi Perempuan		
	Dempo				Lereboleng		
	Anak Krakatau				Ili Boleng		
	**Java**				Ili Werung		
	Salak	Karang	Kiaraberes Gagak		Ili Lewotolo		
	Gede	Pulosar	Perbakti		Sirung		
	Tangkuban Parahu	Patuha	Kawah Manuk		Hobal (sub. volc.)		
	Papandayan	Wayang Windu	Kawah Kamojang		**South Maluku**		
	Guntur	Talaga Bodas	Kawah Karaha		Wetar	Manuk	
	Galunggung	Ungaran			Nieuwerkerk (sub. volc.)	Emp. China (sub. volc.)	
	Ceremai	Merbabu			Wurlali		
	Slamet	Lawu			Teon		
	Dieng	Wilis			Nila		
	Sundoro	Lyang Argapura			Serua		
	Sumbing				Banda Api		
	Merapi			Halmahera Arc	**North Maluku**		
	Kelut				Kie Besi (Makian)	Tokodo	
	Ajurno Welirang				Gamalama		
	Semeru				Gamkonora		
	Bromo				Ibu		
	Lamongan				Dukono		
	Raung			Sangihe Arc	**Sangihe**		
	Kawah Ijen				Colo	Sempu	Tempang
	**Bali-Lombok-Sumbawa**				Ambang	Klabat	Lahendong
	Batur				Mahawu		Tompasu
	Agung				Soputan		Batu Kolok
	Rinjani				Lokon		Sarongsong
	Tambora				Tangkoko		
	Sangeang Api				Ruang		
					Karangetang		
					Banua Wuhu (sub. volc.)		
					Submarine 1922 (sub. volc.)		
					Awu		

## Results

### SO_2_ emission budget

**Fig. 2:**
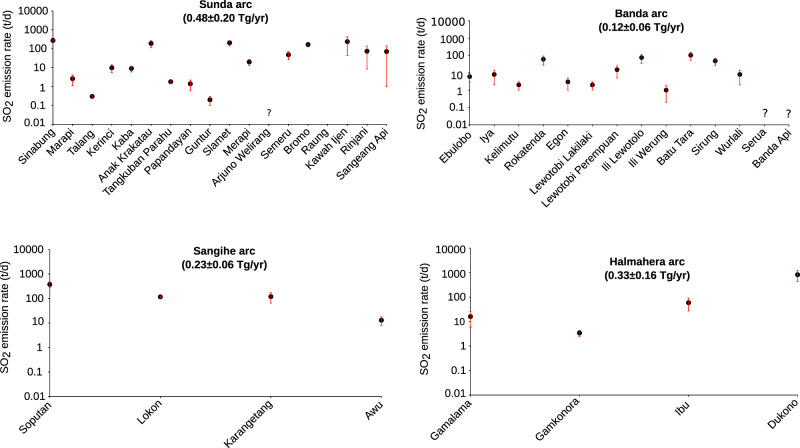
The main volcanic degassing points of Indonesia. The SO_2_ emission rates across the four volcanic arcs of Indonesia highlight the Sunda arc as the largest SO_2_ contributor and Dukono is the strongest individual source. The question marks (?) denote the unmeasured sources and the error bars correspond to standard deviation.

Of the 73 aerial type-A volcanoes across Indonesia, we conducted measurements at 47 (Fig. 1 and Table 3), including 12 that exhibit negligible SO_2_ release. Of the remaining 26 volcanoes that were not visited, 20 are either inactive or exhibit negligible SO_2_ emission, according to local observatory reports and available data and images (https://vsi.esdm.go.id/). There were six volcanoes known for persistent degassing that we did not visit: Banda Api, Serua, Batu Tara, Sangeang Api, Rinjani, and Arjuno Welirang (Fig. 2). However, satellite observations provide some constraints for Batu Tara, Rinjani and Sangeang Api^3^.

**Table 3:** Combined SO_2_ flux results for the Indonesian volcanoes.

Volcano Name	long/lat	Rank	Typical degassing status	Average plume height (m)	Mean SO_2_ flux (Mg/d)	error (Mg/d)	Method/Technique	Source/(measurement date)
**Sunda Arc**								
Sinabung	98.392E/3.170 N	3	emission from lava dome	2600–2800	275	25	DOAS scanner (NOVAC)	Primulyana et al. 2018 (2010–2016)
Marapi	100.474E/0.380 S	29	Minor degassing from the main central crater	2800–2900	2.6	1.5	DOAS Walking traverses	This work (25/10/2014)
Talang	100.681E/0.979 S	35	Minor degassing from fumaroles formed along a NE-SW fracture transecting the summit	2400–2500	0.3	0.04	DOAS Walking traverses	This work (27/06/2012)
Kerinci	101.264E/1.697 S	22	Emission from a lava dome in the crater and frequent eruptions	3200–4300	9.8	4.3	DOAS Walking traverses	This work (03/05/2012)
Kaba	102.615E/3.522 S	23	Degassing of the extended fumarole zone in the crater	1700–1800	9.0	3.1	DOAS Walking traverses	This work (02/06/2015)
Anak Krakatau	105.423E/6.102 S	6	Outgassing through the summit crater filled by a lava flow	400–500	190	77	DOAS Airborne traverses	This work (02/04/2013)
Tangkuban Parahu	107.600E/6.770 S	32	Minor gas release from the main crater	1850–1900	1.8	0.4	DOAS Walking traverses	This work (06/09/2012)
Papandayan	107.730E/7.320 S	33	Hydrothermal dominated gas through three primary fumarolic zones	2250–2300	1.4	0.8	DOAS Walking traverses	Bani et al. 2013 (18/06/2011)
Guntur	107.841E/7.143 S	36	Gas emissions from solfatara	2100–2150	0.2	0.1	DOAS Walking traverses	This work (21/10/2012)
Slamet	109.208E/7.242 S	5	Persistent degassing from the summit crater.	3300–3400	206	66	OMI	Carn et al. 2017 (2005–2015)
Merapi	110.446E/7.540 S	18	Degassing from lava dome	2800–2900	20	7	DOAS scanner	This work (May-Jun.–Jul. 2016)
Arjuno Welirang	112.575E/7.733 S		Degassing associated with vigorous fumaroles—not measured		?			
Semeru	112.922E/8.108 S	16	Continuous eruptive activity with intermittent strong events	3500–3700	48	22	UV-Camera	Smekens et al. 2015 (16–22/05/2013; 31/05–03/06/2013)
Bromo	112.950E/7.942 S	7	Degassing via main crater	2600–2700	166	2	UV-Camera	Aiuppa et al. 2015 (20–21/09/2014)
Kawah Ijen	114.242E/8.058 S	4	Degassing via a solfatara provoked by mining activity	2300–2600	238	194	UV-Camera	This work (12/05/2015)
Rinjani	116.470E/8.420 S	12	Degassing from intracaldera cone	2300–2600	74	65	OMI	Carn et al. 2017 (2005–2015)
Sangeang Api	119.070E/8.200 S	13	Intermittent steam releases	1700–1800	71	75	OMI	Carn et al. 2017 (2005–2015)
				Total SO_2_ for Sunda arc	1313 ± 539 Mg/d (~0.48 ± 0.20 Tg/yr)			
**Sangihe Arc**								
Soputan	124.737E/1.112 N	2	Sustained degassing at the summit and frequent eruptions	1800–2000	376	100	UV-camera	This work (21/07/2014)
Lokon	124.792E/1.358 N	9	Sustained degassing and frequent eruptions	1200–1500	117	10	UV-camera	This work (19/07/2014)
Karangetang	125.407E/2.781 S	8	Degassing associated with lava dome	1700–1900	120	55	DOAS scanner	This work (24/07/2015)
Awu	125.447E/3.689 N	21	Degassing via crater wall and from small intracrater lava dome	900–1100	13	5	DOAS scanner	This work (27/07/2015)
				Total SO_2_ for Sangihe arc	626 ± 170 Mg/d (~0.23 ± 0.06 Tg/yr)			
**Banda Arc**								
Ebulobo	121.191E/8.817 S	26	Degassing via summit fumarolic activity	2100–2300	6	3	UV-Camera	This work (02/10/2014)
Iya	121.641E/8.891 S	24	Degassing via fumarole activity	600–650	8	6	DOAS Walking traverses	This work (01/10/2014)
Kelimutu	121.820E/8.770 S	30	Negligible SO_2_ emission through crater lake	1550–1600	2.0	0.7	DOAS walking traverse	This work (30/09/2014)
Rokatenda (Paluweh)	121.708E/8.320 S	14	Minor gas release via lava dome	700–1000	60	32	OMI	Carn et al. 2017 (2005–2015)
Egon	122.455E/8.676 S	28	Gas release via fumarolic activity at the summit	1600–1700	3	2	UV-Camera	This work (13/05/2013)
Lewotobi Lakilaki	122.768E/8.537 S	31	Minor gas release from summit fumaroles	1350–1400	2.0	0.7	UV-Camera	This work (12/05/2013)
Lewotobi Perempuan	122.781E/8.551 S	20	Degassing from a small intracrater dome	1500–1600	15	10	UV-Camera	This work (12/05/2013)
Ili Lewotolo	123.508E/8.274 S	11	Gas release from the crater and fumaroles	1400–1500	75	40	UV-camera	This work (07/05/2013)
Ili Werung	123.573E/8.532 S	34	Minor gas releases from fumaroles on the flank and crater wall	550–580	1	0.8	UV-Camera	This work (10/05/2013)
Batu Tara	123.585E/7.791 S	10	Degassing from the main crater Eruptive activity: 2012–2015	750–800	102	51	OMI	Carn et al. 2017 (2005–2015)
Sirung	124.130E/8.508 S	17	Degassing via secondary craters. Main crater hosts a crater lake	600–700	48	22	DOAS scanner	This work (13/08/2015)
Wurlali	128.678E/7.125 S	25	Gas release from numerous solfatara zones	700–800	8	6	UV-Camera	This work (21/10/2019)
Serua	130.017E/6.312 S		Minor degassing from the summit		?			
Banda Api	129.881E/4.523 S		Minor degassing from the summit		?			
				Total SO_2_ for Banda arc	330 ± 175 Mg/d (~0.12 ± 0.06 Tg/yr)			
**Halmahera Arc**								
Gamalama	127.330E/0.800 N	19	Degassing via a large fracture at the summit	1650-1700	16	10	DOAS scanner	This work (27/07/2014)
Gamkonora	127.530E/1.380 N	27	Persistent minor degassing	1350-1400	3.4	1.0	DOAS scanner	This work (24/08/2018)
Ibu	127.630E/1.488 N	15	Degassing associated with dome growth and explosions	1200-1300	59	32	DOAS scanner	This work (25/09/2018)
Dukono	127.894E/1.693 N	1	Continuous eruptive activity. with variable intensity	1300-1400	819	394	DOAS scanner	This work (12/07/2015)
				Total SO_2_—for Halmahera arc	897 ± 437 Mg (~0.33 ± 0.16 Tg/yr)			
TOTAL FOR INDONESIA ARCHIPELAGO = 3166 ± 1321 Mg/d (1.15 ± 0.48 Tg/yr)								

Question mark (?) indicates persistently degassing volcanoes not yet measured.

The Sunda arc makes the largest contribution, with a collective daily output of 1313 ± 539 Mg (Fig. 2). Sinabung, Kawah Ijen, Slamet, Anak Krakatau and Bromo volcanoes are the strongest SO_2_ sources of the arc at 275 ± 25 Mg/d, 238 ± 194 Mg/d, 206 ± 66 Mg/d, 190 ± 77 Mg/d, and 166 ± 2 Mg/d, respectively. Moderate to small SO_2_ emission rates have been reported for Rinjani (74 ± 65 Mg/d), Sangeang Api (71 ± 75 Mg/d)^3^, Semeru (48 ± 22 Mg/d)^12^, Merapi (20 ± 7 Mg/d), Kerinci (9.8 ± 4.3 Mg/d) and Kaba (9.0 ± 3.1 Mg/d) (Table 3). These 11 volcanoes are the main degassing sources of the Sunda arc. Measurements have also quantified minor to negligible SO_2_ emission rates (0.2–2.6 Mg/d, Table 3) for five other volcanoes, namely Marapi, Tangkuban Parahu, Papandayan, Talang and Guntur. In total there are 16 volcanic SO_2_ degassing sources across the Sunda arc. Two of them, Dempo and Kelut, host crater lakes that trap condensable gases, limiting their atmospheric contribution. The remaining 19 type A volcanoes out of a total 37 volcanoes across the Sunda are either quiescent (non-emitters) or characterized by low temperature solfataras and/or fumaroles (Table 3), except Ajurno Welirang, which sustains a persistent but minor degassing.

The Banda arc has the lowest passive SO_2_ degassing budget with a total daily output of 330 ± 175 Mg. Ili Lewotolo, Sirung, and Lewotobi Perempuan are the main sources but with moderate emission rates corresponding to 75 ± 40 Mg/d, 48 ± 22 Mg/d, 15 ± 10 Mg/d. Batu Tara and Rokatenda with 102 ± 51 and 60 Mg/d, respectively^3^, are among the main SO_2_ degassing sources of the Banda arc. Note that the figure for Batu Tara was obtained by subtracting our measurement for Ili Lewotolo from the reported combined flux for both volcanoes^3^. Lesser SO_2_ emission rates are found for Iya (8 ± 6 Mg/d), Wurlali, (8 ± 6 Mg/d) and Ebulobo (6 ± 3 Mg/d). We obtain negligible fluxes for Egon (3 ± 2 Mg/d), Kelimutu (2.0 ± 0.7 Mg/d), Lewotobi Lakilaki (2.0 ± 0.7 Mg/d), and Ili Werung (1.0 ± 0.8 Mg/d) (Table 3). Two other volcanoes of the Banda arc, including Serua, and Banda Api, were not visited and therefore their SO_2_ emissions remain unknown. However, based on information from the local observatories, degassing strength of Banda Api is comparable to that of Wurlali, and exceeds that of Serua. The lack of measurements is thus unlikely to bias significantly our arc-scale flux estimate. The SO_2_ degassing associated with low temperature solftaras and fumaroles from the remaining eight type A volcanoes of this arc is negligible (Table 3).

The Sangihe arc hosts three volcanoes with relatively strong SO_2_ emission rates, including Soputan (376 ± 100 Mg/d), Lokon (117 ± 10 Mg/d) and Karangetang (120 ± 55 Mg/d). Awu the northernmost volcano of the arc emits 13 ± 5 Mg SO_2_/d. The SO_2_ contribution from the five remaining volcanoes is negligible (Table 4). Hence, with a total SO_2_ degassing budget of 626 ± 170 Mg per day, the Sangihe arc constitutes a notable arc-scale volcanic degassing source to the atmosphere. Note, however, that Soputan clearly stands out as the strongest source, representing 60% of the total for this arc.

**Table 4:** Number of eruptive events per year and the corresponding SO_2_ release per arc for 2010–2020 period for both from satellite and VEI results.

	2010	2011	2012	2013	2014	2015	2016	2017	2018	2019	2020	Total events/arc	Mean number of events/yr
Number of eruptive events													
Sunda	5	4	2	6	9	6	3	8	8	6	3	60	5
Banda	0	0	4	3	2	2	0	1	0	0	1	13	1
Sangihe	1	3	3	1	1	3	2	1	2	1	1	19	2
Halmahera	1	2	2	2	2	2	2	1	2	1	1	18	2
Total events/year	7	9	11	12	14	13	7	11	12	8	6	110	10
SO_2_ emission per arc (Tg)												Total SO_2_ (Tg)	Mean Tg/yr
Sunda	0.172	0.050	0.009	0.004	0.574	0.006	0.047	0.000	0.021	0.028	0.002	1.069 ± 0.184	0.097 ± 0.011
	0.641	0.044	0.022	0.082	0.824	0.235	0.056	0.151	0.821	0.722	0.118	3.715 ± 0.337	0.337 ± 0.021
Banda	0.000	0.000	0.001	0.005	0.000	0.000	0.000	0.000	0.000	0.000	0.062	0.068 ± 0.019	0.006 ± 0.003
	0.000	0.000	0.136	0.103	0.037	0.022	0.000	0.004	0.000	0.000	0.095	0.397 ± 0.05	0.036 ± 0.008
Sangihe	0.000	0.010	0.003	0.000	0.000	0.001	0.000	0.000	0.014	0.005	0.000	0.035 ± 0.005	0.003 ± 0.002
	0.095	0.133	0.133	0.018	0.004	0.118	0.099	0.004	0.114	0.017	0.019	0.755 ± 0.055	0.069 ± 0.018
Halmahera	0.061	0.060	0.063	0.047	0.099	0.080	0.203	0.104	0.055	0.248	0.407	0.143 ± 0.011	0.013 ± 0.003
	0.095	0.114	0.099	0.099	0.114	0.114	0.099	0.095	0.099	0.095	0.095	1.121 ± 0.008	0.102 ± 0.033
Total Tg/yr	0.233	0.121	0.075	0.057	0.673	0.088	0.250	0.104	0.090	0.281	0.473	1.314 ± 0.180	0.119 ± 0.045
	0.832	0.291	0.390	0.302	0.979	0.489	0.254	0.253	1.034	0.835	0.327	5.988 ± 0.310	0.544 ± 0.116

Underlined values are those obtained from VEIs.

Finally, the SO_2_ emission rate from the Halmahera arc amounts to 897 ± 437 Mg/d with more than 90% of this flux accounted for by Dukono which emits 819 ± 394 Mg/d. The other volcanoes are low to moderate sources, with 59 ± 32 Mg/d from Ibu, 16 ± 10 Mg/d from Gamalama and 3.4 ± 1.0 Mg/d from Gamkonora. On Kie Besi, only a small fumarole is present and its SO_2_ emission is considered negligible.

Based on the above results, and as summarized in Table 3, the total daily SO_2_ emission passively released into the atmosphere from the entire Indonesian archipelago is 3200 ± 1300 Mg/d, equivalent to 1.15 ± 0.48 Tg SO_2_ yr^−1^ (Table 3). We emphasise that this figure is representative of the periods of observations and must be viewed cautiously but we believe it gives a useful guide to the scale of emissions at the scale of the entire archipelago.

### Principal point sources

Our ranking of Indonesian volcanic sources of passive degassing is shown in Table 3. Dukono (Halmahera arc) is the strongest, representing more than a quarter (26%) of the total. Soputan (Sangihe arc), Sinabung and Kawah Ijen (Sunda arc) are also notable, representing, 12%, 9% and 8% of the total, respectively. These four volcanoes alone constitute around half of the total inventory. They are sustained by different magma compositions, i.e., basaltic andesite to andesite on Sinabung^13^, basaltic to dacite on Kawah Ijen, basaltic on Soputan^14^, and andesite to trachyandesite on Dukono^15^. Six other volcanoes exhibit moderate rates of SO_2_ emission, including Slamet (206 Mg/d), Anak Krakatau (190 Mg/d), Bromo (166 Mg/d), Karangetang (120 Mg/d), Lokon (117 Mg/d) and Batu Tara (102 Mg/d). Thus, ten volcanoes contribute 82% of the total passive volcanic SO_2_ emission budget of Indonesia. There are five other volcanoes with modest SO_2_ fluxes, between 50 and 100 Mg/d that together represent 11% of the budget, seven with SO_2_ emission rates between 10 and 50 Mg/d, representing 5% of the budget, and finally 14 volcanoes whose SO_2_ degassing is below 10 Mg/d.

### Arc scale variations

Our new SO_2_ inventory reveals substantial variations in SO_2_ output between the arcs of the Indonesian archipelago. The 3000-km-long Sunda arc, with 37 type A volcanoes, is the largest SO_2_ source at 0.48 ± 0.20 Tg/yr representing 41% of the Indonesian total. The 2000-km-long Banda arc, in contrast, contributes just 0.12 ± 0.06 Tg/yr representing only 10% of the total, despite hosting 24 Type A volcanoes. The 600-km-long Sangihe and 500-km-long Halmahera arcs are stronger sources (0.23 ± 0.06 Tg and 0.33 ± 0.16 Tg SO_2_ yr^−1^, respectively) despite their shorter extents and comparatively few volcanoes (eleven and five, respectively). It is thus plausible that the geodynamic contexts play a key role in the SO_2_ emission budget of Indonesia; in terms of SO_2_ emission rate per km of arc, the Halmahera arc is the strongest source with an output of 655 Mg SO_2_ per km yr^−1^ followed by the Sangihe, Sunda and Banda arcs with 380, 160 and 60 Mg SO_2_ per km yr^−1^, respectively.

### Passive and explosive degassing

**Fig. 3:**
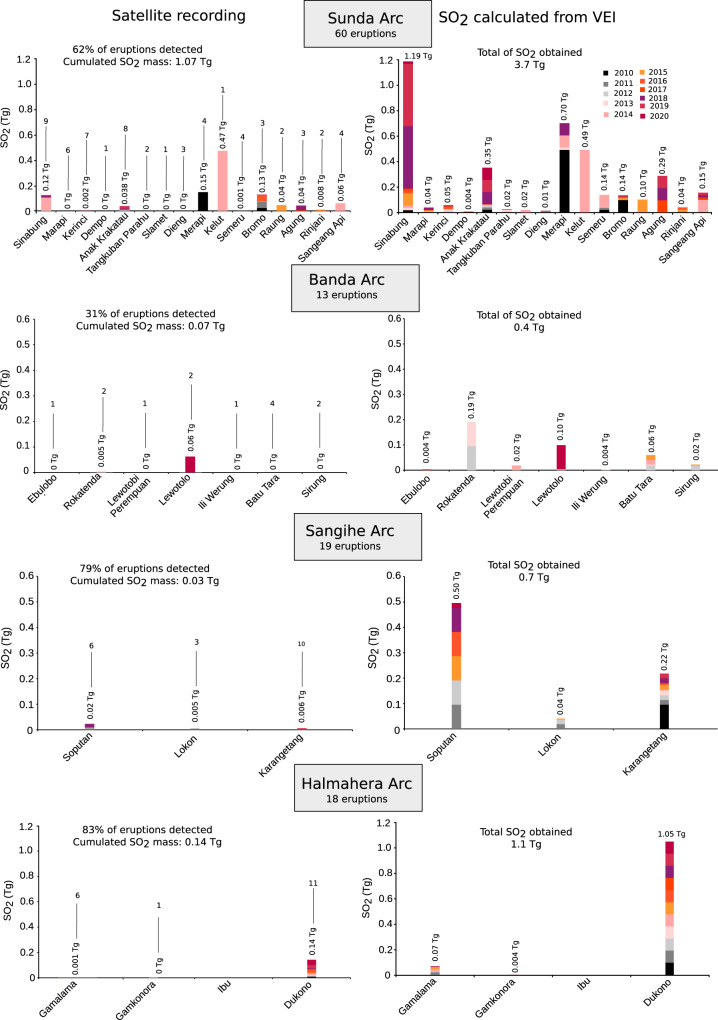
The explosive SO_2_ released per volcano over the period of 2010–2020. The names of the volcanoes that erupted over the decade are grouped by arc. The SO_2_ mass per volcano obtained from satellite data are displayed on the left column whilst the right column shows the SO_2_ amount obtained from the VEIs. The 0 Tg correspond to undetected eruptive emission by satellite sensors. The color code differentiates the years of observation and the height corresponds to the amount of SO_2_ released per year. The number of eruptions per volcano is provided above each SO_2_ mass value on the left column. Note that Dukono exhibits a continuous eruptive manifestation but only the largest event with ash fall on the nearby cities are considered.

Our total Indonesian SO_2_ inventory of 1.15 ± 0.48 Tg/yr based on ground-based and airborne surveys of persistent volcanic degassing across the archipelago is half the estimated emission from the 20 Indonesian volcanoes reported in ref. ^3^, derived from satellite remote sensing measurements. But this latter approach focused on a different study period (2005–2015) and integrated the SO_2_ contribution from explosive events. Between 2010 and 2020, there were 110 eruptive episodes across the Indonesian archipelago reported in the Global Volcanism Program (https://volcano.si.edu/) and Bandan Geologi (https://vsi.esdm.go.id/). Most of these were minor to moderate in scale (VEI < 3) and their SO_2_ emissions were, mostly, not captured by satellite sensors. The year 2014 was the most active year with 14 eruptions while in 2020 there were only six eruptive events reported. The mean value is ten eruptions per year (Table 4 and Fig. 3). The Sunda arc dominates this list with 60 eruptions at 16 different volcanoes. Sinabung and Anak Krakatau were the most active with nine and eight events, respectively. Kerinci and Marapi were also notably active with, respectively, six and seven eruptions, whilst Merapi, Semeru, and Sangeang Api experienced four eruptions each. The Banda arc produced 13 eruptions at seven different volcanoes over the last decade. Batu Tara was the most active with four eruptions. For the Sangihe arc, 19 eruptions were reported at three different volcanoes, including Karangetang, the most active with ten eruptions, Soputan with six eruptions and Lokon with three eruptions. Eighteen eruptive events were recorded for the Halmahera arc. Dukono was the most active volcano with 11 eruptions, followed by Gamalama with six eruptions.

To estimate the SO_2_ contribution from these explosive events, we first used a formulation^16^ relating volcanic explosivity index (VEI) and SO_2_ yield:

1 $${{\log }}10({{{\rm{SO}}}}_{2},{{{\rm{Tg}}}})=0.71{{{\rm{VEI}}}}-3.15$$

We took VEI values reported in the Global Volcanism Program (https://volcano.si.edu/). This indicates a total eruptive SO_2_ output over the 2010–2020 period of 5.99 ± 0.31 Tg with annual totals varying between 0.25 Tg and 1.03 Tg (Table 4, Fig. 3). The Sunda arc released 3.7 ± 0.3 Tg, representing 62% of the total, with the main contributions from Sinabung, Merapi, Kelut, Anak Krakatau, and Agung. For the Banda arc we estimate 0.40 ± 0.05 Tg SO_2_ (0.04 Tg/yr) accounting for 7% of the budget, mostly contributed by Rokatenda, Lewotolo and Batu Tara. For the Sangihe arc, Soputan, Karangetang and Lokon volcanoes were the only sources with an estimated combined yield of 0.75 ± 0.05 Tg (0.07 Tg/yr) of SO_2_ or 12% of the total. Lastly, the Halmahera arc released 1.12 ± 0.01 Tg (0.10 Tg/yr) of SO_2_ through eruptions, representing 19% of the total. Dukono was the main contributor, accounting for 83% of the arc’s output (Table 4).

We also analyzed available satellite data (http://SO2.gsfc.nasa.gov/) for the SO_2_ mass over Indonesia between 2010 and 2020. Out of the 110 eruptions, 71 were captured by the satellite (64%) and the corresponding SO_2_ mass amounts to a total of 1.31 ± 0.18 Tg, with a mean annual value of 0.12 ± 0.04 Tg. The Sunda and Halmahera arcs are the main contributors representing, respectively, 81% (1.07 ± 0.18 Tg) and 11% (0.14 ± 0.01 Tg) of the total SO_2_. The main contributors from the Sunda arc are Kelut (44%), owing to its 2014 eruption^17^, Merapi (14%), primarily related to its 2010 event^18^, and Sinabung (12%), which has experienced episodic dome growth and collapse since 2010 (ref. ^19^). For the Halmahera arc, 99% of the arc contribution is from Dukono, reflecting its continuous eruptive activity. For the Sangihe arc, the total SO_2_ yield amounts to 0.03 ± 0.005 Tg, and Soputan, with its recurrent eruptive activity, is the main source, representing 67% of the arc contribution. Finally, the Banda arc contribution corresponds to 0.068 ± 0.019 Tg and is dominated by the 2020 eruption of Ili Lewotolo, representing 92% of the arc contribution. These figures obtained from satellite data are lower than those calculated from the VEIs (Fig. 3 and Table 4), except for Kelut and Ili Lewotolo, where the estimates converge. Despite the discrepancy, particularly in the lower SO_2_ estimates, both approaches highlight the Sunda and Halmahera arcs as the main SO_2_ contributors to explosive emissions.

**Fig. 4:**
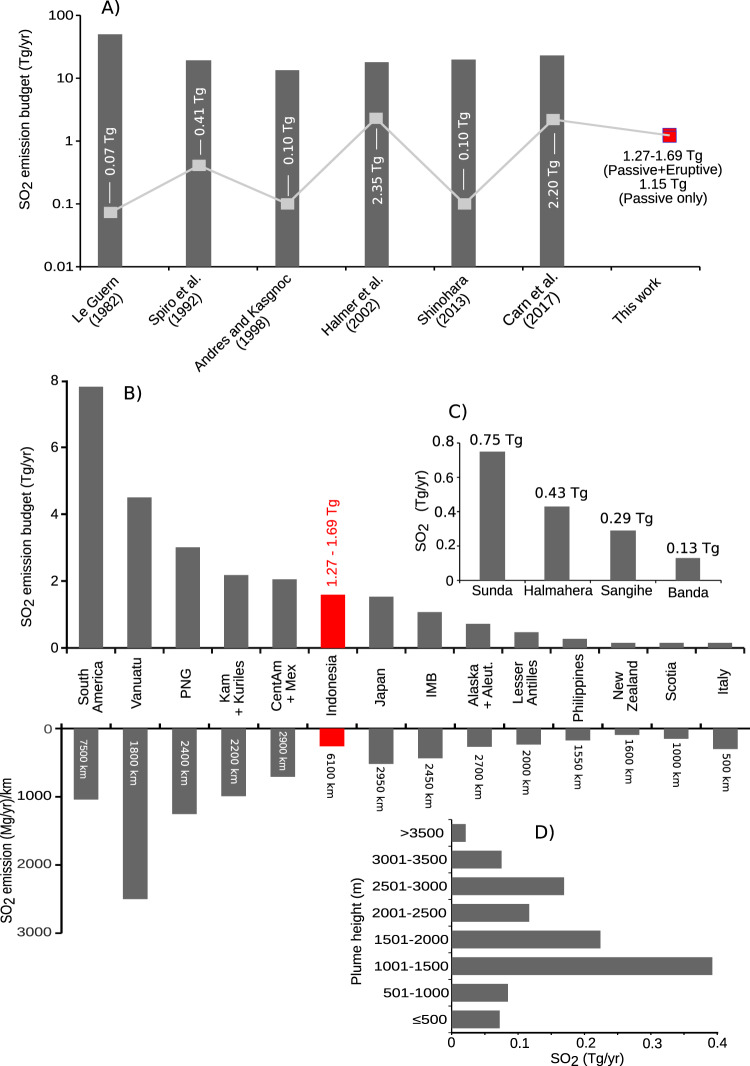
The new SO_2_ flux results compared to other estimates. **A** Estimates of the global volcanic SO_2_ inventory that include contributions from Indonesian volcanoes, highlighted by gray square with the corresponding values. **B** The Indonesian SO_2_ emission budget compared with other arcs (data from ref. ^20^). The annual SO_2_ emission per km of each arc^21^ are shown for comparison. **C** The SO_2_ emission budgets from the four Indonesian arcs. **D** Strength of passive SO_2_ emissions by altitude (in 500-m bins) from the observations reported in Table 3.

Combining the figures we derive for passive and explosive SO_2_ degassing yields a total source between 1.27 and 1.69 Tg SO_2_ /yr for the Indonesia archipelago. The lower and the higher range integrate respectively the mean annual figures from satellite data and from the VEIs (Table 4). About 10% to 30% of total SO_2_ release was sustained by larger, sporadic explosive emissions. This Indonesia total budget corresponds to 3–7% of the global volcanic SO_2_ emission budget based on estimates in ref. ^3^ and^20^ (23–33 Tg/yr) and is comparable to the total emissions from Japan^21^ (Fig. 4), although much less when the degassing budget is normalized by arc length.

## Discussion

This work constitutes the first near-comprehensive SO_2_ emission survey across the Indonesian archipelago. We estimate a passive degassing flux of 1.15 Tg SO_2_/yr for Indonesia in the period of 2010–2019. This represents the cumulative emission from twenty volcanoes, the four strongest sources being Dukono, Soputan, Sinabung and Kawah Ijen, which together represent 54% of the total emission budget, while Slamet, Anak Krakatau, Bromo, Karangetang, and Lokon account for another 25% of the total. More modest sources include Batu Tara, Ili Lewotolo, Rinjani, Sangeang Api, Rokatenda, and Ibu, representing 14% of the total. Seven minor sources, Semeru, Slamet, Merapi, Gamalama, Lewotobi Perempuan and Awu account for the remainder. We have also estimated explosive SO_2_ emissions from Indonesia for the period 2010–2020 based on a simple scaling from reported VEI values and satellite records. The mean annual explosive-SO_2_ obtained range between 0.12 and 0.54 Tg/yr, 63–81% of which is associated with the Sunda arc (0.10–0.34 Tg/yr), 5–7% (0.04–0.07 Tg/yr) the Banda arc, 3–13% (0.03–0.07 Tg/yr) the Sangihe arc and 11–18% (0.10–0.14 Tg/yr) the Halmahera arc. Combining the calculated passive and explosive SO_2_ data suggests a total volcanic SO_2_ yield for the Indonesian archipelago of 1.27–1.69 Tg/yr. We consider this a representative figure, acknowledging that it is based on very limited temporal sampling of the volcanoes in question.

Our SO_2_ inventory indicates a surprisingly modest SO_2_ emission budget for Indonesian volcanoes, considering the 6000 km extent of the archipelago, four distinct volcanic arcs, 126 active volcanoes, and on the order of ten larger eruptions per year. For comparison, Ambrym (Vanuatu) and Kīlauea (Hawaii) volcanoes alone have passively released more SO_2_ into the atmosphere: 2.7 Tg/yr and 1.8 Tg/yr, respectively^3^. Several individual eruptions of the last 15 years also released comparable or higher SO_2_ amounts compared with the annual Indonesian output, including Kasatochi (2.7 Tg) in 2008, Sarychev Peak (1.2 Tg) in 2009, Eyjafjallajökull (1.2 Tg) in 2010, and Nabro (4.5 Tg) in 2011^16^. This modest SO_2_ emission budget also contrasts with the picture of renowned climate-changing Indonesian eruptions, including Agung 1963 (ref. ^22^), Tambora 1815 (ref. ^23^), Krakatau 1883 (ref. ^24^), and Samalas 1257 (ref. ^25^). More recently, the Galunggung eruption of 1982–1983 (Java) yielded 2.5 Tg of SO_2_ (ref. ^26^).

While the overall SO_2_ budget is unremarkable, the bulk of the emissions are into the free troposphere (Fig. 4D) likely to extend timescales of atmospheric processing and deposition of sulfur^27^. Previous studies have highlighted the contribution of sulfur deposition from volcanic plume to sulfur emissions from peat fires^28^.

Factors controlling sulfur output are numerous and include deep source characteristics and chemical processes occurring during magma storage and transfer through the crust. Hydrothermal scavenging and scrubbing of sulfur from magmatic-hydrothermal discharges is often invoked as a process for sulfur depletion in volcanic fluids, significantly altering the magmatic signature^29^. Acidic crater lakes, which are numerous in Indonesia, are perhaps the most obvious manifestation of such processes^30^. Substantial sulfur deposits are known to be sequestered by volcanic lake systems and, conceivably, variations in climatic conditions, notably rainfall, across the archipelago could play a role in volcanic emissions to the atmosphere.

**Fig. 5:**
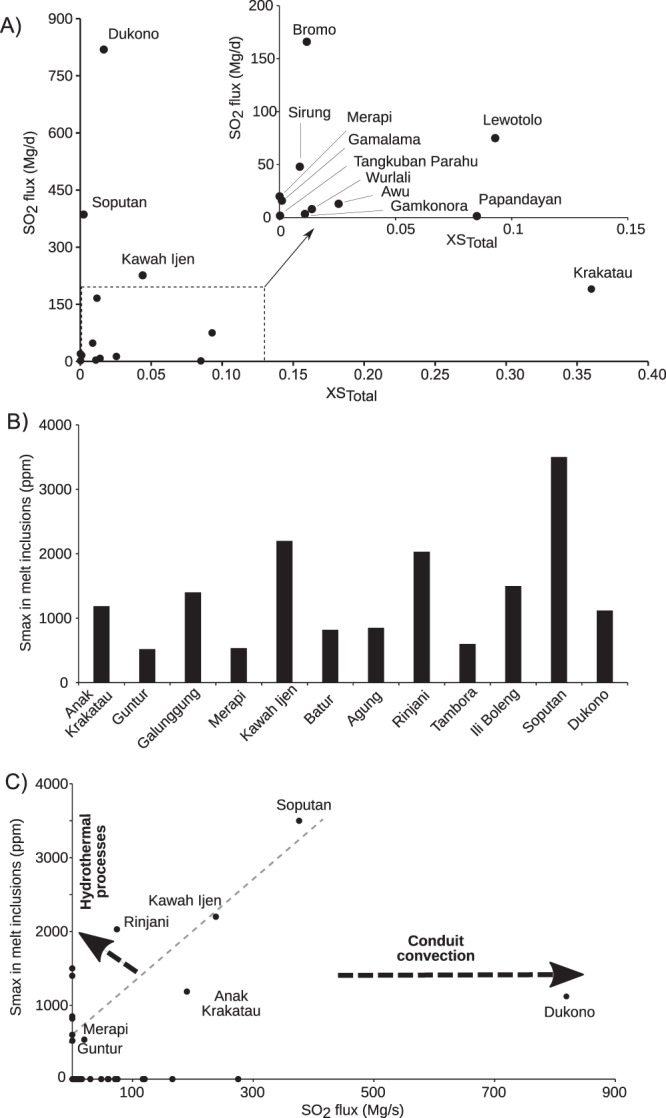
Sulfur content in fluids and melt inclusions versus SO_2_ fluxes. **A** Variation of the sulfur content of volcanic fluids (XS_Total_ = XSO_2_ + XH_2_S) with the measured SO_2_ flux along the Indonesian arc. References for fluid compositions are from Allard et al. 1981^56^ (Krakatau); Poorter al. 1989^57^ (Lowotolo); Giggenbach et al. 2001^58^ (Merapi, Tangkuban Parahu, Papandayan); Clor et al. 2005^59^ (Soputan); Aiuppa et al. 2015^60^ (Bromo); Gunawan et al. 2017^61^ (Kawah Ijen); Bani et al. 2017^62^ (Sirung); Bani et al. 2017^15^ (Dukono); Saing et al. 2020^63^ (Gamkonora); Kunrat et al. 2020^64^ (Gamalama); Bani et al. 2020^65^ (Awu). **B** The record of sulfur in melt inclusions along the Indonesian arc in respect to the main degassing sources. **C** Relationships between the maximum sulfur content analysed in melt inclusions (MI) and the measured SO_2_ flux. Note, we did not find melt inclusion values for data points with zero SO_2_ flux and conversely. References for MI are: Vidal et al., 2016^25^ (Rinjani); Mandeville et al., 1996^66^ (Krakatau); Bani et al., 2017^15^ (Dukono); Preece et al., 2014^67^ (Merapi); de Hoog et al. 2001^68^ (Guntur, Ili Boleng); Vigouroux et al. 2012^69^ (Kawah Ijen, Galunggung, Tambora); Self and King, 1996^22^ (Agung); Self et al. 2004^70^ (Tambora), Kunrat, 2017^41^ (Soputan).

The subaerial sulfur output will depend on initial gas composition, the flow path, gas-wall rock heat transfer, and the effective water to rock ratio, all parameters that are difficult to constrain and which vary greatly between volcanoes. However, were hydrothermal scavenging and scrubbing leading mechanisms, one would expect flux strength to correlate with the concentration of sulfur in fluids. Instead, for the few volcanic centres for which, in addition to SO_2_ flux, there are constraints on H_2_O, CO_2_, H_2_S + SO_2_ species in fumaroles, we find no correlation between the mass fraction of sulfur in the fluid, XS_tot_ (=XSO_2_ + XH_2_S) and SO_2_ flux (Fig. 5A), which presumably is a function of both the initial volatile content of the magma and degassing conditions. Dukono’s gas, in particular, does not have higher sulfur than other Indonesian fumaroles (the case of Krakatau needs to be confirmed by more measurements). This indicates that SO_2_ flux need not reflect particular enrichment/depletion in sulfur of the emitted gas, and implicates the role of degassing vigour during the time interval considered (which can scale with conduit radius and presence of an open vent to the atmosphere). From this perspective, it is worth emphasising that to establish robust links between volcanic degassing and processes at depth, it requires comprehensive measurement of gas composition.

While hydrothermal sequestration of sulfur is likely to play a significant role in modulating subaerial emissions, we consider also whether arc scale differences in SO_2_ emissions across the Indonesian archipelago might reflect geodynamic or source controls, as proposed for CO_2_ in arc magmas worldwide^31^. The amount of SO_2_ released per km of arc per year reveals the Halmahera arc as the strongest SO_2_ source, followed by the Sangihe arc. The magmatic sources of these two arcs are sustained by the double subduction of the Molucca Sea plate that deepens to the west beneath the Sangihe arc, and to the east under the Halmahera arc^15^. Geochemistry of lavas sampled along these arcs indicates enriched magma sources in fluid-mobile elements and notable sediment contributions^32,33^, which may play a role in subaerial sulfur budgets. The steepening of the subducted slab, the downward force from the Philippine Sea plate, and the westward motion of the continental fragments along the Sorong fault could have promoted fluid fluxes into the mantle wedge along the Halmahera arc^15^. To a first approximation, this peculiar geodynamic context may explain elevated SO_2_ fluxes at both Halmahera and Sangihe arcs simply because of enhanced magmatic activity.

In contrast, the Banda arc stretches 2000 km but exhibits a remarkably low SO_2_ emission, the weakest in our inventory. The arc is also characterized by anomalously low ^3^He/^4^He ratios^34^ reflecting the arc collision with the Australian continental block and subduction of continental material that ultimately supplies less sulfur to the mantle wedge, compared to subduction of oceanic plate^35^.

The Sunda arc is the largest SO_2_ source, representing 43–48% of the total, however, its annual SO_2_ emission per km of arc is modest compared with the Halmahera and Sangihe arcs, and with other arcs worldwide^20^. The magma source beneath the Sunda arc is sustained by subduction of the Indo-Australian plate. However, while deep sea drilling has revealed a 1400 m sediment column in front of Sumatra, 300 m in front of Java, and 500 m in front of Sumbawa^36^, less than 15% of these sediment columns is subducted^37^. The mass transfer along the Sunda arc is dominated by an active frontal accretionary prism that strongly limits sediment subduction. Each year only 2.6 × 10^7^ m^3^ of sediment is subducted beneath the Sunda arc, compared with the 1.8 × 10^8^ m^3^/yr available, given the average subduction speed of 6.7 cm/yr. Furthermore, the sediment input from the Sunda arc is mostly trapped in the forearc basin and does not reach the trench. Given that subducted sediment can strongly contribute to the volcanic sulfur budget, it is possible that this active accretional prism plays a key role in modulating the SO_2_ emission budget of the Sunda arc by limiting the mass transfer of sediment-derived sulfur into the mantle wedge. However, as shown in the next section, such a variability in sediment contribution is not evident in variable sulfur abundance in magmas sustaining Indonesian arc volcanism.

Arc volcanoes are typically supplied by reservoirs in the shallow crust, which are in turn fed by basaltic melts rising from the mantle wedge and carrying an imprint of slab volatiles. Because they are hot, these mafic magmas have a higher sulfur carrying capacity than cooler silicic magmas, hence any volcano erupting mafic magmas should generally be associated with stronger sulfur emissions (though of course arc-scale variations in mafic melt sulfur content are possible). Detailed petrological studies of Indonesian volcanoes remain scarce, and only a few have had their sulfur content characterized via analysis of melt inclusions (MI). Figure 5B draws on these studies and shows the highest sulfur contents measured in MI along the Indonesian arcs. Drawing rough relationships from these data, basaltic MI have 2000–3500 ppm S, andesitic MI 800–500 ppm S, and rhyodacitic 200–300 ppm S, with no obvious geographical trends along arc being apparent, assuming that primary sulfur contents in the melt inclusions are little affected by post entrapment processes.

The most evident feature is that Dukono, the strongest SO_2_ source we identify, has basaltic melt inclusions with rather low S content (1000 ppm) relative to other, currently weaker SO_2_ sources, such as Rinjani or Kawah Ijen, which have MI with sulfur in excess of 2000 ppm. Similarly, Soputan emits half the SO_2_ of Dukono, yet has basaltic MI with much higher sulfur content (3500 ppm). This is illustrated in Fig. 5C, which shows that a broad positive correlation between SO_2_ flux and the maximum sulfur content, S_max_, in MI. Only Dukono departs significantly from the trend indicated by other centres. This again argues for strong decoupling between the fertility of the immediate source of magma degassing (the crustal reservoir) and its ultimate surface manifestation. Such a behavior may simply reflect conduit dynamics, such as convection, which is strongly dependent on conduit radius^38–40^, and which can sustain strong SO_2_ degassing of an otherwise comparatively sulfur-poor reservoir. Alternatively, a more sulfur-rich magma may give rise to a low sulfur output simply because of a low magma influx that cannot sustain conduit convection. A critical parameter is the conduit radius, *R*, since magma (and gas) flux scales with *R*^4^ (ref. ^40^) such that small variations in conduit size can result in large fluctuation in sulfur flux.

The composition and temperature of the magma supplied to the conduit will also be important, owing to their influence on viscosity and rheology. Hotter and fluid material will promote not only higher rates of magma overturn in the conduit but also more efficient degassing of slowly diffusing species, such as sulfur, in silicate melts. From this perspective, systems lying above the dashed line toward Soputan, whose high SO_2_ flux may be considered as directly related to its basaltic magma source^14^ and high sulfur content in MI^41^ (Fig. 5C), may reflect more pronounced scavenging by the aquifer/hydrothermal system overlying magma reservoirs, limiting sulfur emissions to the atmosphere. These local controls will be superimposed on any deeper source signatures and may even obliterate them, as exemplified here by Dukono.

The SO_2_ emission budget of the Indonesian archipelago thus reflects a complex interplay between deep (geodynamic) factors that control primary magma compositions and their availability along the arc and superficial processes such as hydrothermal scavenging and conduit dynamics. Reservoirs regularly supplied by fresh hot magma may promote sustained and vigorous degassing via conduit convection, leading to strong SO_2_ outputs even when sulfur complements in the melt are comparatively poor. In other words, the vigour of convection may largely compensate for, or even offset, any deep source deficiency in sulfur. The relatively low SO_2_ output for the Indonesian archipelago documented here may appear in stark contrast with the record of explosive eruptions at several Indonesian volcanoes and their recognized global climate impacts (e.g. Tambora, Krakatau, Rinjani). These events, however, essentially reflect the long term accumulation of magmas and volatiles in closed crustal reservoirs, which cool and fractionate with little volatile loss, a process that differs from the persistent or passive degassing operating at open-conduit systems such as those we document here. An in depth knowledge of the petrology of volcanic products, and a robust characterization of emanating fluids, are both required if sound connections between the plumbing system and degassing are to be established at any active volcano.

## Methods

### Passive ultraviolet spectrometers

We used two techniques, USB-controlled ultraviolet spectrometers and Differential Optical Absorption Spectroscopy (DOAS)^42^ and ultraviolet cameras (UV-cam)^6^. The passive ultraviolet spectrometers were either carried beneath the plume on a moving platform^43^ or located in a fixed position and attached to scanning optics^7^. Both approaches yield the SO_2_ profile across the plume. The spectrometers used were the Ocean Optics USB2000 (280–400 nm, 0.5 nm FWHM resolution), USB4000 (290–440 nm, and 0.3 nm FWHM), and USB2000 + (290–440 nm and 0.5 nm). For traverse measurements, the spectrometer was connected via an optic fibre bundle to a vertically pointed telescope of 8 mrad FOV (Field Of View). The location of each recorded spectrum was obtained using a continuously recording GPS unit. The DOAS traverse setup requires no additional power supply since the spectrometer is powered by the laptop. We operated the equipment onboard a light aircraft, from a 4WD vehicle, and on foot.

For the scanning observations, we used a rotating window that accepts light from selected directions across the plume. The light that transits through the window is redirected to an embedded telescope by a 45° optical prism, then transmitted to the spectrometer via optical fibre. The rotating window was attached to a stepper motor controlled by the laptop via a microcontroller. The system was designed to perform a 180° scanning angle with a minimum step angle of 1.8°. The scanner required an external 12 V power supply. The scanning setup could be readily operated by one person. Spectra were acquired using Jscript executed by DOASIS software^44^. The script used in both traverse and scanning allowed optimization of the signal-to-noise ratio by automatically adjusting exposure time and numbers of co-added spectra^45^. This was particularly useful for scanning, given the change of light intensity with scan angle. Both traverse and stationary recording were carried out at distance varying between few tens of meters from the craters to around 5 km downwind, depending on the access difficulties, the plume size and the volcanic activity.

SO_2_ column amounts (ppm m) were retrieved using standard DOAS calibration and analysis procedures outlined in ref. ^43^. Reference spectra included in the non-linear fit were obtained by convolving high-resolution SO_2_ and O_3_ cross-sections with the instrument line shape. A Fraunhofer reference spectrum and ring spectrum, calculated in DOASIS, were also included in the fit. The optimum fitting windows were selected where they provided a near-random fit residual with minimum deviation. The total SO_2_ column amount across the plume was then multiplied by the estimated plume speed to obtain the SO_2_ flux. The plume velocities were measured mainly using videography and handheld anemometers, except in the case of airborne measurements where the plume speed was obtained by flying along and against the plume axis.

### Ultraviolet cameras

The imaging setup consisted of two Apogee Alta U260 UV cameras. Each was coupled to a Pentax B2528-UV lens, with a focal length of 25 mm allowing a full angle FOV of around 24°. Immediately in front of each lens, a 10 nm (FWHM) bandpass filter was placed, one filter was centered at 310 nm (Asahi Spectra XBPA310) where SO_2_ absorbs and the other at 330 nm (Asahi Spectra XBPA330) outside the SO_2_ absorption region. Image acquisition and processing were achieved using Vulcamera^46^. For each pixel the optical depth (OD) was obtained according to the following equation:

${{{\rm{OD}}}}=-{{{\rm{ln}}}}\{[({{{\rm{PA}}}}-{{{\rm{DA}}}})/({{{\rm{CA}}}}-{{{\rm{DA}}}})]/[{{{\rm{PB}}}}-{{{\rm{DB}}}}/({{{\rm{CB}}}}-{{{\rm{DB}}}})]\}$ where A and B represent the camera with the 310 nm and 330 nm filters respectively, and P, D, and C represent plume, dark and clear images. To correlate the OD values with the SO_2_ slant column densities (SCDs), four calibration cells with known amounts of SO_2_ (94, 189, 475, 982 ppm.m) were used. Calibration images were acquired at the beginning of measurements and repeated with long series of measurements. The UV-cam was generally positioned with a view perpendicular to the plume transport direction, at distance between <1 km and 6 km depending on plume size. Plume speeds were derived during data processing by following plume structures between two fixed lines perpendicular to plume transport direction^46^.

### Uncertainties

Uncertainties in DOAS and UV-cam SO_2_ flux measurements are discussed in many past works, including the following ref. ^47^. The dominant error in the retrieved SO_2_ column amount is induced by the variability of light intensity and the distance between the plume and instruments. With increasing distance, light that has not traveled through the plume may contribute significantly to the signal. This leads to light dilution of the plume signal that can easily cause more than 50% underestimate in SO_2_ emission rate^48^. To reduce this effect in the UV-cam measurement, we deployed the system during clear sky conditions, at distances <6 km, and performed calibration every hour during long series of measurements. UV-cam measurements were performed mainly in the late morning before the clouds started to formed, generally at 9–11 am.

For the DOAS measurement, we compensate for light intensity changes using an artificial constant dark, calculated from each recorded spectrum, in the ‘UV blind’ region (below 290 nm). Such corrections account for dark spectrum, offset and stray light. We estimate that the error in the column amount contributes ~0.01 to the squared variation coefficient of the total flux. We also assumed that the plume and transport direction is homogeneous and in a straight line since it is difficult to rigorously assess in this work. We, therefore, performed flux calculations for direction ϕ, ϕ±3, and ϕ±6. The mean contribution to the square variation of the total flux is in the order of a thousandth. These errors are negligible in comparison to uncertainties in the plume speed that resulted from the complexity of wind field around volcanoes and frequent variations in both time and space. The plume transport speed relative error is conservatively assumed to be about 30–35%, which is towards the higher end of the range of past estimates^49^. These errors are applied to each traverse and profile then the mean value is calculated for each series of measurement with the corresponding standard deviation. The global estimates for the arc is the sum of the mean values.

## Supplementary information

Peer Review File

## Data Availability

The data that support the plots within this article are available from the corresponding author upon reasonable request.
